# Exploring Anatomical Variations of Abdominal Arteries Through Computed Tomography: Classification, Prevalence and Implications

**DOI:** 10.7759/cureus.41380

**Published:** 2023-07-04

**Authors:** Basil Jalamneh, Ismael J Nassar, Leen Sabbooba, Raya Ghanem, Zaher Nazzal, Ruba Kiwan, Ahmed Awadghanem, Mosab Maree

**Affiliations:** 1 Faculty of Medicine and Health Sciences, An-Najah National University, Nablus, PSE; 2 Department of Radiology, Al-Essra Hospital, Amman, JOR; 3 Department of General Practice, Palestinian Ministry of Health, Ramallah, PSE; 4 Department of Dermatology, King Abdullah University Hospital, Ar-Ramtha, JOR; 5 Department of Community and Family Medicine, An-Najah National University, Nablus, PSE; 6 Department of Medical Imaging, Health Science North, Northern Ontario School of Medicine Sudbury, Ontario, CAN; 7 Department of Radiology, An-Najah National University Hospital, Nablus, PSE; 8 Department of Medicine, College of Medicine and Health Sciences, An-Najah National University, Nablus, PSE

**Keywords:** accessory, replaced, embryological mechanisms, celiac trunk, hepatic artery, renal artery, aorta, computed tomography, anatomical variation

## Abstract

Background and aims: Variations in the branches of the abdominal aorta are relatively prevalent and can impact certain surgeries. The accurate identification and differentiation of these variations pre- and intraoperatively are crucial to avoid negative clinical sequelae. This study aimed to investigate the prevalence of variations in some branches of the abdominal aorta and to identify the most frequent variants as well as any rare variants not previously classified in the existing classification systems. The study's findings may help improve the understanding and management of these variations.

Materials and methods: This retrospective study was conducted at the Department of Radiology at An-Najah National University Hospital (NNUH) and included 550 abdominal computed tomography (CT) angiographic scans for patients (51.5% males, 48.5% females) performed between January 2017 and January 2023.

Results: Variations were most common in the hepatic arteries (34.7%), followed by the renal arteries (31.3%). Variations in the celiac trunk were the least frequent (9.8%). The gastro-splenic trunk (type V) was the most common celiac trunk variant. The most common hepatic artery variant was the replacement of the right hepatic artery (type III). Accessory renal arteries were more frequent on the left side and among males (P = 0.01). The celiac trunk variations had a significant association with the hepatic artery variations (P = 0.001) and the renal artery variations (P = 0.011), respectively.

Conclusion: There is a high prevalence of anatomical variations in the described vessels, and it matches the results in the reported literature. Our findings also suggest the possible coexistence of variants. We have also encountered rare variants, especially in the hepatic arterial system. Some of the hepatic arterial system variants are not included in the older classification systems, calling for an extension of the old systems (Michel’s and Hiatt classification systems) or replacement with the newer (CRL or EX-CRL classification systems) to account for rare variants not previously classified. Radiologists and surgeons should be proficient in identifying and differentiating these variations to take precautions and actions for each variant individually.

## Introduction

During embryologic development, the digestive tube differentiates into foregut, midgut, and hindgut. These three parts are all supplied by the abdominal aorta (AA) that branches ventrally into the celiac trunk, the superior mesenteric artery (SMA), and the inferior mesenteric artery (IMA). The celiac trunk supplies the foregut structures, it branches into the common hepatic artery (CHA), the splenic artery (SA), and the left gastric artery (LGA). Classically, the CHA divides into right and left branches to supply each liver lobe. The stomach is supplied by all three celiac trunk branches that anastomose at their lesser and greater curvatures [1]. In general, the gastrointestinal (GI) vascular system is known for its anastomoses at many levels. Moreover, variations in the branches of these arteries are common.

Uflacker et al. described seven variants in the celiac trunk [2], and Michel et al. described 10 variants of hepatic vascular anatomy in their study on cadavers [3]. Variations in the renal artery include the presence of an additional renal artery that has its separate aortic ostium, called an accessory renal artery [4].

We aimed to identify these previously classified variations and any unclassified variants among the Palestinian population and report their frequencies. Moreover, we reviewed the related embryologic theories and reviewed some of the variants' potential clinical implications.

## Materials and methods

Study design and study sample

This is a descriptive retrospective cross-sectional study. It has been carried out, after ethical approval from the NNU institutional review board (IRB), in the Department of Radiology at An-Najah National University Hospital (NNUH). Informed consent was not required as the study was conducted on secondary data without personal identification. NNUH is a Joint Commission International (JCI)-accredited multidisciplinary tertiary teaching hospital, which receives omnifarious types of cases referred from all over the country. We examined images of abdominal computed tomography (CT) scans with intravenous (IV) contrast from our hospital picture archiving and communication system (PACS).

CT protocol

CT images were examined using 128 multidetector CT scans (SOMATOM, SIEMENS Healthineers, Erlangen, Germany), with slice thickness range of 0.6-7mm, a voltage of 120-140 kV, and 180-220 mA current, 0.33 s/rotation helical system. Nonionic low osmolar omnipaque contrast was used and injected by test bolus or bolus tracking technique at a rate of 2ml/s-6ml/s, using Medrad Stellant dual-head injector (Medrad, Pennsylvania, USA).

CT examination

We examined 558 CT scans from the archive of patients who were referred to the NNUH Radiology Department between January 2017 and January 2023. Two consultant radiologists read images independently. Unique findings were revised twice. Multiplanar reconstruction (MPR) images of axial, coronal, and sagittal plans and 3D reconstruction images of CT were used for evaluation to get precise results. We included images for patients with documented demographic factors and past medical and surgical history. The images included are high-quality, with apparent vascular anatomy and no obscuration of the normal vascular anatomy due to any factor, such as insufficient contrast, previous surgical manipulation of vessels, or tumor effect.

We excluded eight images that did not satisfy our inclusion criteria (response rate of 98.56%); six of which were low-quality images with insufficient IV contrast resulting in difficult examination and anatomy identification. Other images were for a patient who had a large hepatic tumor that obscured the accurate examination of the target vessels and a patient with previous surgery that disturbed the normal vascular anatomy, respectively.

Study variables

**Figure 1:**
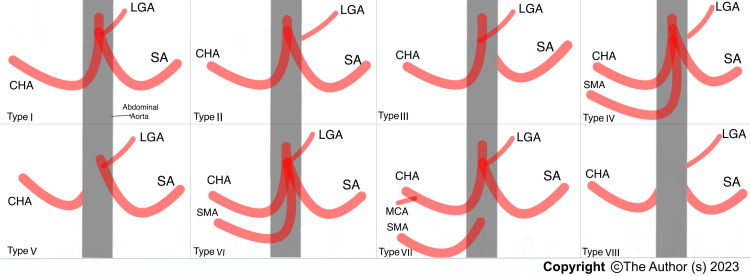
Uflacker’s classification for celiac trunk variations. Type I = Hepatogastrosplenic trunk, type II = Hepatosplenic trunk, type III = Hepatogastric trunk, type IV = Hepatosplenicmesenteric trunk, type V = Gastrosplenic trunk, type VI = Celiac-mesenteric trunk, type VII = Celiac-colic trunk, type VIII = no celiac trunk. CHA: common hepatic artery, LGA: left gastric artery, SA: splenic artery, SMA: superior mesenteric artery, MCA: middle colic artery

Anatomical variations of the celiac trunk were described according to Uflacker’s classification (Figure 1) [2].

**Figure 2:**
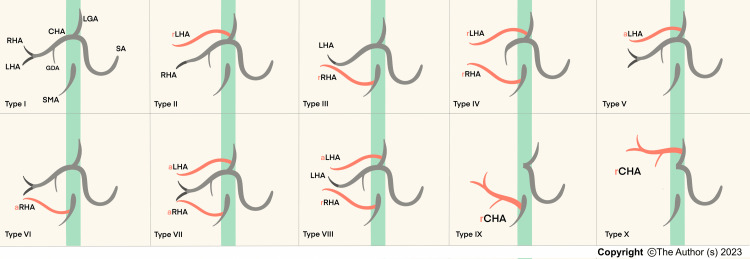
Hepatic artery variations according to the Michel's classification. Type I = typical pattern. Type II and III = Replaced left and right hepatic arteries, respectively. Type V and VI = Accessory left and right hepatic arteries, respectively. Type IV = type II + type III. Type VII = type V + type VI. Type VIII = type III + type V. Type IX CHA that arises from the SMA. Type X = CHA arises from the LGA. CHA: common hepatic artery, LHA: left haptic artery, RHA: right hepatic artery, a: accessory, r: replaced

Anatomical variations of the hepatic arteries were defined according to Michel’s classification (Figure 2) [3]. Hiatt’s classification system for hepatic artery variation is also reported in the literature. Hiatt’s classification described only six variants, and it is the simplified version of Michel’s classification system [4]. However, we chose the broader Michel’s classification system to be more inclusive. Type I is the typical anatomical pattern in all previously mentioned classification systems [2,3].

The renal artery classically arises from the lateral side of the abdominal aorta below the superior mesenteric artery origin. The presence of an additional renal artery is called an accessory renal artery [4]. We observed the whole path of the artery, any artery that enters the renal hilum, lower or upper pole and has its separate aortic ostium was regarded as an accessory artery.

These variants were described in various terms in literature, such as anomalous, aberrant, replaced, or accessory. However, one should differentiate a replaced artery from an accessory artery. Replaced means that the original artery does not exist at all, while accessory indicates the presence of an additional artery along with the original one [5].

Statistical analysis

The data were analyzed using Statistical Product and Service Solutions (SPSS) (IBM SPSS Statistics for Windows, Armonk, NY). The frequency and percentage of each variable were calculated. We used the Chi-square test to investigate the relationship between the arteries variants. The significance level was set to P-value <0.05.

## Results

The study included 550 CT scans with IV contrast for patients aged between three and 95 years, with an average age of 48 years; 51.5% were males and 48.5% were females.

Celiac trunk

**Figure 3:**
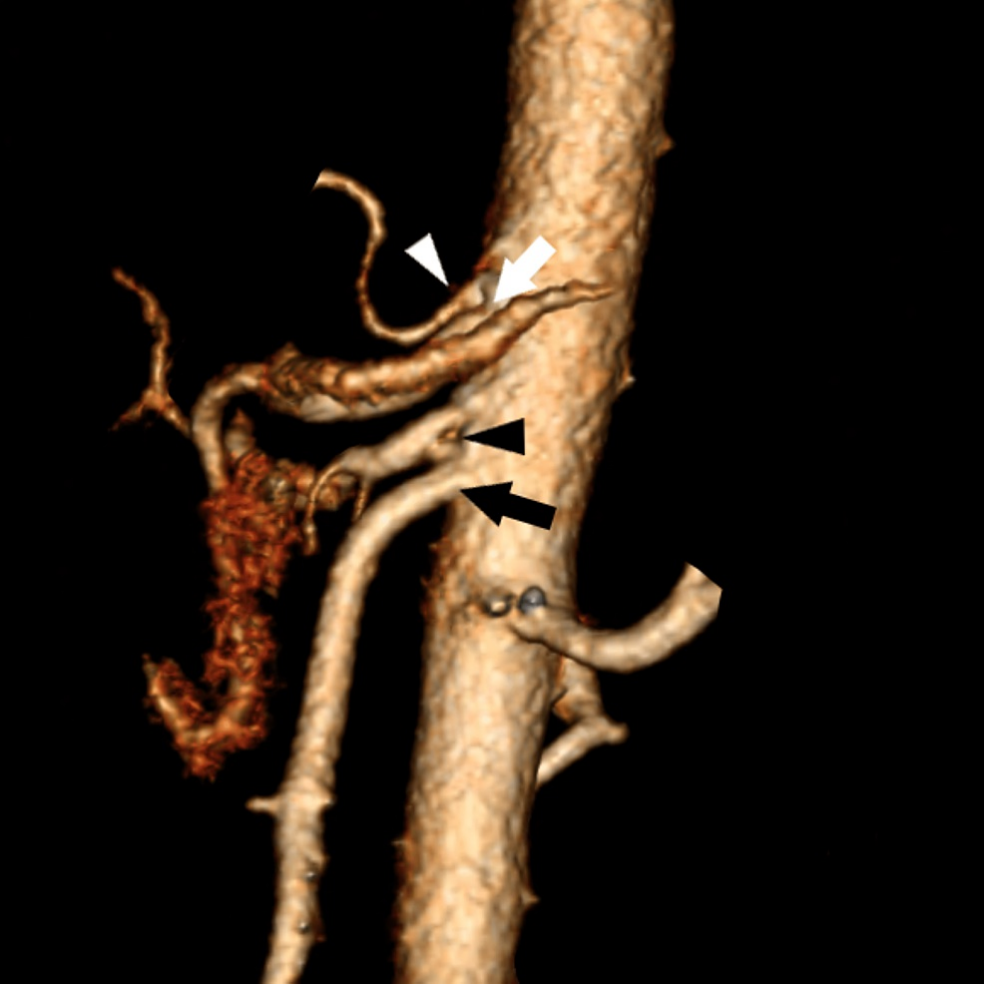
Sagittal view with a 3D reconstruction that shows celiac trunk and hepatic artery variants: (1) Type II celiac trunk variant (white arrowhead). And (2) the associated hepatic variants; replaced the right hepatic artery (black arrowhead) that arises directly from AA. White arrow = celiac trunk, black arrow = SMA. AA: abdominal aorta, SMA: superior mesenteric artery

**Figure 4:**
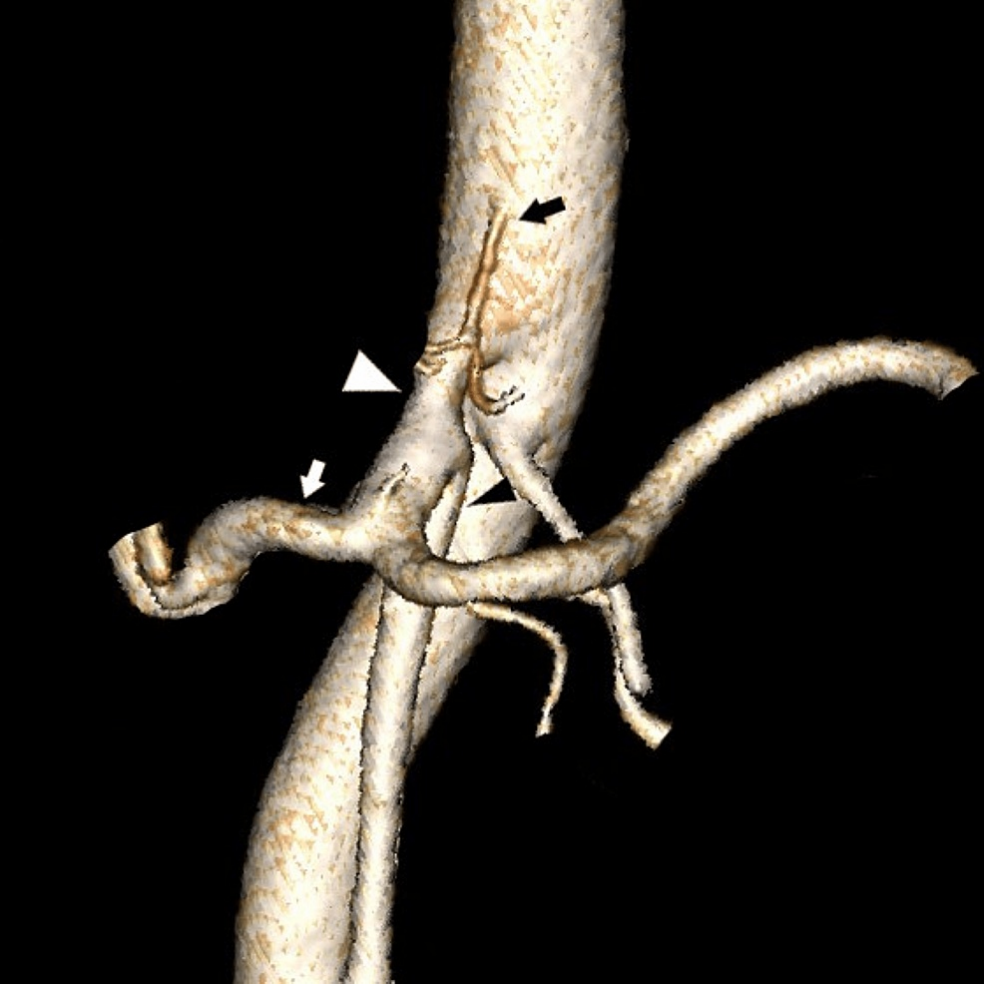
This 3D reconstruction image shows the type IV variant according to Uflacker’s classification, where SMA (black arrowhead) arises within the celiac trunk (white arrowhead) and LGA (black arrow) arises directly from AA. White arrow = CHA. SMA: superior mesenteric artery, LGA: left gastric artery, AA: abdominal aorta, CHA: common hepatic artery

**Figure 5:**
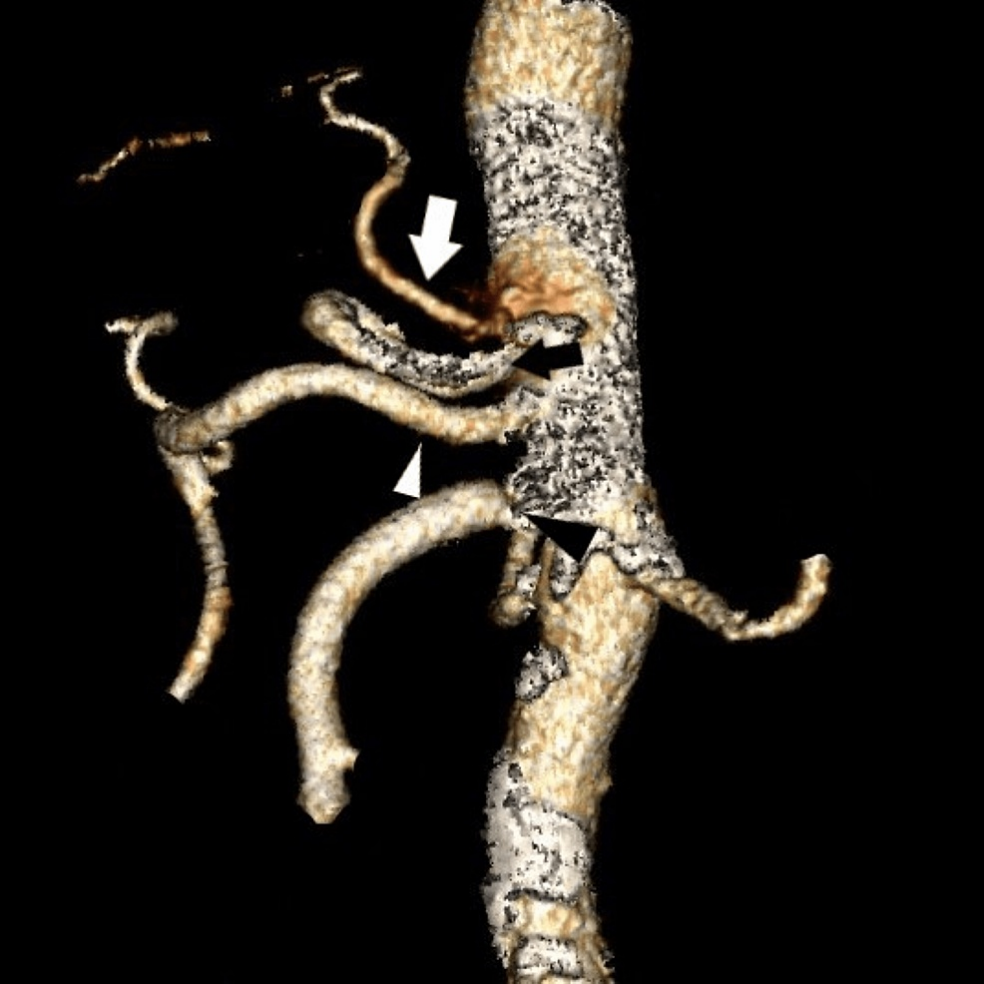
This sagittal 3D reconstruction image of type VIII Uflacker’s classification shows the absence of the celiac trunk; each branch arises directly from AA; white arrow = LGA, black arrow = SA, white arrowhead = CHA, black arrowhead = SMA. AA: abdominal aorta, LGA: left gastric artery, SA: splenic artery, CHA: common hepatic artery, SMA: superior mesenteric artery

The anatomical variations in the celiac trunk pattern were observed in 54 cases (9.8%). Among the variants, type V was the most common (5.5%), which is a gastrosplenic trunk with the CHA originating directly from the AA or SMA. More variants are listed in Table 1. Type II, type IV, and type VIII variants encountered in our study are illustrated in Figure 3, Figure 4, and Figure 5, respectively.

**Table 1:** Number of cases and percentages of celiac trunk variations (n=550). ‡ see Figure 3, * see Figure 4, † see Figure 5

Celiac trunk classification (Uflacker)	Number of cases	Percentage
Type I	496	90.2%
Type II ‡	17	3.1%
Type III	0	0
Type IV*	1	0.2%
Type V	30	5.5%
Type VI	1	0.2%
Type VII	0	0
Type VIII †	5	0.9%

Hepatic artery

We found variations in the hepatic arterial system in 191 cases (34.7%). The most common variant was type III (8.4%). Other variants that are not listed in Michel’s classification have been identified in our study (Table 2). A significant relationship between celiac trunk and hepatic artery variations was established (P=0.001) (Table 3).

**Table 2:** Number of cases and percentages of hepatic artery variants (n=550). * Replaced left hepatic artery with accessory right hepatic artery. † Replaced left hepatic artery associated with CHA that arises from the superior mesenteric artery. ‡ Accessory left hepatic associated with CHA that arises from the superior mesenteric artery. NOC: Not otherwise classified (in Michele’s classification system) CHA: common hepatic artery, AA: abdominal aorta

Hepatic artery classification (Michel’s types)	Number of cases	Percentage
Type I	359	65.3
Type II	44	8.0
Type III	46	8.4
Type IV	15	2.7
Type V	38	6.9
Type VI	11	2.0
Type VII	5	0.9
Type VIII	4	0.7
Type IX	19	3.5
Type X	0	0
Type II with accessory right hepatic artery arising from the celiac trunk (NOC)	1	0.2
Coexistence of Type II and VI (NOC)	2	0.4
Replaced right hepatic artery arises directly from AA; see Figure 3 (NOC)	3	0.5
Coexistence of Type IX and Type II † (NOC)	1	0.2
Coexistence of Type IX and Type V ‡ (NOC)	1	0.2
Accessory right hepatic artery arises directly from the celiac trunk (NOC)	1	0.2

**Table 3:** The frequencies of all studied arterial systems and their correlation with gender and with each other.

Variations	Gender		P-value*
	Male	Female	
Hepatic artery variation			0.096
Absent	194 (68.8%)	156 (61.8%)	
Present	89 (31.2%)	102 (38.2%)	
Celiac trunk variation			0.728
Absent	254 (89.8%)	242 (90.6%)	
Present	29 (10.2%)	25 (9.4%)	
Renal artery variation			0.010
Absent	181 (64.0%)	198 (74.2%)	
Present	102 (36.0%)	69 (25.8%)	
Variations	Hepatic artery variation		P-value*
	Absent	Present	
Celiac trunk variation			0.001
Absent	335 (93.3%)	161 (84.3%)	
Present	24 (6.7%)	30 (15.7%)	
Renal artery variation			0.513
Absent	244 (68.0%)	135 (70.7%)	
Present	115 (32.0%)	56 (29.3%)	
Variations	Renal artery variation		P-value*
	Absent	Absent	
Celiac trunk variation			0.011
Absent	350 (92.3%)	146 (85.4%)	
Present	29 (7.7%)	25 (14.6.7%)	

Renal artery

**Figure 6:**
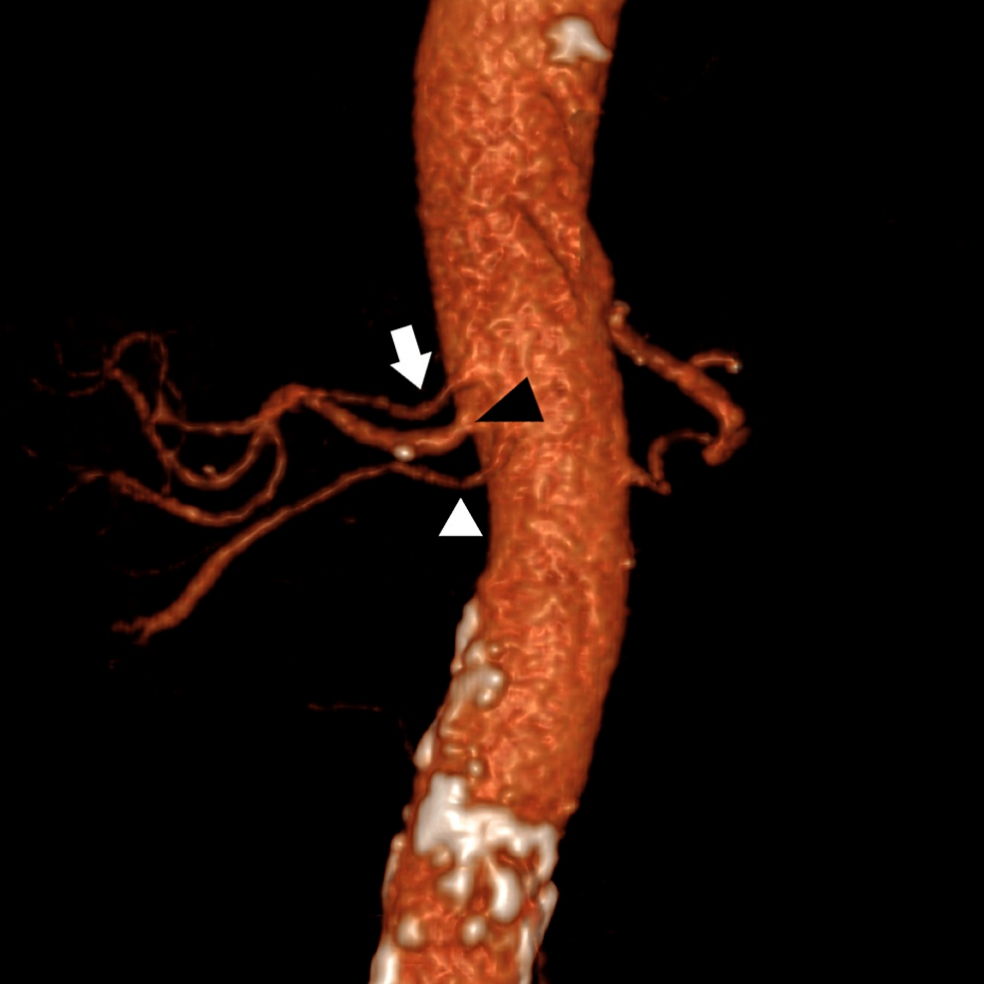
A 3D reconstruction image that shows two right accessory renal arteries in the upper and lower kidney poles (white arrow and white arrowhead, respectively). Black arrowhead = Main right renal artery.

Accessory renal arteries were found in 31.3% of the cases. The most prevalent variant was the left accessory renal artery (12.9%), other variants are described in Table 4, these include one, two, or three accessory renal arteries that are found either on the left, the right, or both sides. Figure 6 shows two right accessory renal arteries. In addition, the accessory renal artery was significantly more common among males (P=0.010) and correlates significantly with celiac trunk variants (P=0.011) (Table 4).

**Table 4:** Number of cases and percentages of renal artery variants.

Renal artery accessory	Number of cases	Percentage
No accessory artery	379	68.9
Left accessory artery	71	12.9
Right accessory artery	56	10.2
Bilateral accessory arteries	31	5.6
Two left accessories and one right accessory artery	2	0.4
Two right accessories and one left accessory artery	1	0.2
Two bilateral accessory arteries	2	0.4
Two left accessory arteries	4	0.7
Two right accessory arteries*	3	0.5
Three left accessories arteries	1	0.2

## Discussion

Variations in the abdominal aorta branches are not uncommon, the high prevalence of variations in our study as well as previous studies should raise concerns about the importance of its identification preoperatively or intraoperatively and its possible clinical sequelae.

The celiac trunk, which is also known as the celiac axis or the celiac artery, is a major branch of the abdominal aorta that arises from its ventral surface at the level of the diaphragm or T12 vertebra. It divides into three main branches. One is the common hepatic artery which constitutes the major arterial blood supply of the liver. The common hepatic artery gives rise to the gastroduodenal artery the proper hepatic artery. The proper hepatic artery enters the liver and branches subsequently for each liver lobe. The right hepatic artery supplies the right liver lobe and the left hepatic artery supplies the left liver lobe. Further small branches arise from the right and left hepatic arteries. The cystic artery arises from the right hepatic artery to supply the gallbladder [6].

In this study, our choice to examine some of the major abdominal vessels together (the celiac trunk, the hepatic artery, and the renal arteries) was not only because of their clinical importance but also to highlight any correlation between them in light of the proposed embryologic mechanisms. The initial fetal vascular anatomy is different from the adult, many vessels may develop initially and then regress. In general, vascular variations are the product of failed vascular regression, resulting in a new atypical anatomy [7].

During embryonic development, the descending aorta is established through the fusion of the two embryonic dorsal aortae located caudal to the pharyngeal arches. Subsequently, the abdominal aorta is formed, giving rise to three distinct groups of arteries. The first group consists of dorsal arteries, which traverse posteriorly to the spinal cord and the body wall, forming the lumbar arteries. The second group is lateral arteries bilaterally, namely the suprarenal, renal, and gonadal arteries. Among these, the renal arteries are the largest. Conversely, the third group encompasses the anterior arteries, which supply the gut. This group includes the celiac trunk, supplying the foregut, the superior mesenteric artery, supplying the midgut, and the inferior mesenteric artery, supplying the hindgut [8-10].

The celiac trunk, the superior mesenteric artery, and the inferior mesenteric artery arise from the ventral surface of the abdominal aorta at different levels, the roots are connected by a longitudinal anastomosis that may regress (separating the vessels and resulting in normal anatomy) or persist (giving rise to the variations) [7,11].

The kidneys develop initially in the pelvis and as they ascend, they receive new vessels from the lateral side of the AA that sprouts at different successive levels. The primary vessels should degenerate as the kidneys go to their final location. When these vessels persist, it becomes accessory renal arteries [12,13].

Celiac trunk

The prevalence of variations in this artery was reported in close frequencies in the literature [14,15]. Santos et al. systemic review of 12 articles presented the prevalence of variations in the celiac trunk from 9% to 38% [16]. The naming system of the variants can be confusing; however, one can relate more by indicating the name of the arteries that form the variants, the typical celiac trunk is a trifurcation named gastro-hepato-splenic trunk (i.e., the hepato-gastric trunk is an atypical bifurcation instead of the trifurcation, formed only by the hepatic artery and gastric artery, with the splenic artery originating from the abdominal aorta directly).

Therefore, a combination of any two of the three common branches can result in a bifurcation that is either the gastro-splenic trunk, the hepato-gastric trunk, or the hepato-splenic trunk. Bifurcation was the most common variant in our study as well as in Santos et al. regardless of its subtype [16]. We found the type V bifurcation pattern (the gastrosplenic trunk) as the most prevalent, a result matching several studies [6,14].

The hepatosplenomesentric trunk (type IV) is formed when the celiac trunk branches to the SMA, SA, and CHA, the LGA arises directly from the AA. We found one case of this variant as well in Arifuzzaman et al. and Ugurel et al. [6,14].

When the SMA or the middle colic artery (MCA) arises from the celiac trunk in addition to its classical three branches, a quadrifurcation of the celiaco-mesenteric trunk (type VI) or the celiocolic trunk (type VII) will be formed, respectively. Although these are rare variants, we found one case of type VI in our study.

By referring back to the embryological theory, when complete regression of the ventral vessels occurs, the total absence of the celiac trunk is seen (type VIII). We found five cases of this variant in our study, and Santos et al. described it in five reviewed articles [16].

Hepatic artery

Compared to the celiac trunk, variations are more common in the hepatic arteries, the variations prevalence was reported as 48% in Ugurel et al. and 45% in Binit Sureka et al. [6,17], our results of the variation (34.7%) are close to Gumus et al. (33.2%) [18], and Arifuzzaman et al. (30.9%) [14].

The replaced right hepatic artery (type III) was reported as the most frequent in many studies [6,18]. While another study reported replacement of both the right and the left hepatic artery (type IV) as the most frequent [14], we found it only in 2.7% of cases.

We found some variants that do not fit in Michel’s classification of the hepatic artery variants, these include six types described in (Table 2). Fortunately, another newer and wider classification system is suggested in the literature, called the “CRL classification system", which classified all the rare variants, such as the ones found in our study, and was not included in Michel’s classification system [19].

However, Xiaojing Wu et al. reported a case of a hepatic artery arising from the phrenic artery that does not fit in the CRL classification system either and suggested a modified classification system (EX-CRL classification) [20].

Preoperative identification of anatomical variations is valuable clinically, especially with the increased reliance on laparoscopic and minimally invasive procedures, that have limited visualization in the surgical site. While some variations are benign and do not have a surgical value, others do. Dealing with the encountered variations should be individual. The complications should be anticipated according to the surgical site, the type of surgery, and the type of anatomical vascular aberrancy. The complications appear to be essentially due to direct damage of the aberrant vessel leading to intraoperative bleeding or indirect damage of an organ due to hypoperfusion resulting in ischemia and possible necrosis if a tissue depends solely on the aberrant vessel that has been cut or ligated [11]. Moreover, an incomplete understanding of the vascular complexity associated with the presence of these variations can extend the operation time [10,21].

In laparoscopic-assisted gastrectomy procedures, the left gastric artery origin and branches should be assessed accurately, the presence of a replaced hepatic artery originating directly from the left gastric artery could expose an erroneous liver injury [22].

Identification of this aberrant anatomy can overcome an iatrogenic hypoperfusion injury to the liver, by ligating the left gastric artery distal to the origin of the replaced hepatic artery and closer to the stomach curvature [16,22]. The identification of the left gastric artery origin is also helpful in catheterization procedures to diagnose and control gastric hemorrhage [23].

Nevertheless, the variations in the hepatic arteries are important in most hepatobiliary surgeries as well as pancreatic surgeries. Each variant has its clinical impact. In pancreaticoduodenectomy (PD), which typically has a high rate of iatrogenic liver injuries (10%) [24], the presence of a replaced hepatic artery can affect its course around the pancreas (posterior or anterior to the pancreas, pre-pancreatic or post-pancreatic). It was also found that a replaced right hepatic artery poses an increased risk of complications of liver ischemia, necrosis, and abscess. On the other hand, replaced left hepatic artery or accessory hepatic artery poses no risk. However, both preoperative identification and intraoperative visualization of the variants can expand the surgeon’s awareness of the possible consequences and impact his decision to perform a reconstruction procedure when necessary [10,25].

Other implications of hepatic arterial system variations include liver transplantation [26], intraarterial chemotherapy, and radioembolization of the liver [11,22,27].

However, we did not describe the course of the aberrant anatomical variants nor the complete evaluation of the hepatobiliary system including the cystic artery that would be helpful in laparoscopic cholecystectomy procedures [5]. We recommend including a wider inclusive study that examines all the abdominal vasculature.

Renal artery

Different frequencies are reported in studies held in different regions. Gulas et al. analyzed renal artery variations in 28 different ethnic groups, the frequency of variation ranged from as low as 4% [28] to 59.5% [4].

We found renal artery variations in 31.1% of cases. The variants are an additional renal artery (named accessory artery) present most commonly on the left, as well as the right or both sides. Two left accessory renal arteries are also described by Ugurel et al. [6]. We found two accessory renal arteries on the left, the right, and both sides. We also found one rare case of three left accessory renal arteries (see Table 3).

Several studies have consistently reported the predominance of renal artery variations on the left side [6,14]. Similarly, it is widely reported in the literature that kidney malformations are commonly found on the left side. This prevalence discordance could be attributed to the asymmetric development of the kidneys as well as each kidney's unique properties such as location, size, volume, and anatomic relations. The ascent of the kidney on the right side is restricted by the liver, leading to a left kidney located higher than the right kidney. The aorta is located slightly left to the midline, while the inferior vena cava is positioned right to the midline, this may favor left accessory renal vessels development and impair its development on the right. The specific mechanisms responsible for these lateralizations require further investigations, but they may involve vascular development, differential gene expression, or susceptibility to environmental factors such as hypoxia [29].

Despite calling it an accessory renal artery - which appears to be a misleading term - a whole segment of the kidney may rely on it. Therefore, attention should be granted during surgical interventions and ligations, since this can lead to irreversible ischemia in the corresponding kidney part [6]. One example is infrarenal abdominal aortic aneurysm repair, Sadeghi-Azandaryani et al. concluded that a decrease in renal function following endovascular aneurysm repair was more pronounced in patients with accessory renal arteries. The presence of an accessory renal artery also decreases the transplantation success rate [30-32].

Variations in the renal artery were more prevalent among males in our study. We also found that the renal artery variations correlate significantly with the celiac trunk variations but it does not correlate with the hepatic artery variations. From the few studies that included the three arterial systems as in ours, Ugurel et al. reported a similar significant correlation [6]. We could not explain the exact mechanism of this correlation embryologically, but one can conclude that variants may coexist. That being said, it is essential to thoroughly examine the whole gastrointestinal vascular anatomy when encountering any variation.

Although our study was a single-center study, we found several rare variants, due to a relatively large number of cases.

## Conclusions

In conclusion, our study shows a high prevalence of variations in the abdominal aorta branches. These findings emphasize the value of identifying and differentiating these variations preoperatively or intraoperatively to avoid possible clinical sequelae. It is crucial to understand the embryological mechanisms of the formation of these variations to correlate between the variants. Our study examined some major branches of the abdominal aorta to highlight any correlation between them.

Our results suggest a significant relationship firstly between celiac trunk and hepatic artery variations, and secondly between the renal artery and hepatic artery variations; this indicates the possible coexistence of the variants. Furthermore, our study found some rare variations that are not present in the older classification systems. Therefore, the existing classification systems may be updated or expanded to account for these rare variants. Some variants are benign, others have an apparent clinical impact. We recommend that future studies focus more on the clinical implications of each variant individually. In addition, radiologists and surgeons should be proficient in identifying and differentiating these variations to ensure optimal patient care.
